# Effects of Enamel Matrix Derivative on Cell Spheroids Made of Stem Cells Obtained from the Gingiva on Osteogenic Differentiation

**DOI:** 10.3390/medicina59020377

**Published:** 2023-02-15

**Authors:** Somyeong Hwa, Hyun-Jin Lee, Youngkyung Ko, Jun-Beom Park

**Affiliations:** 1 Department of Periodontics, College of Medicine, The Catholic University of Korea, Seoul 06591, Republic of Korea; 2 Dental Implantology, Graduate School of Clinical Dental Science, The Catholic University of Korea, Seoul 06591, Republic of Korea

**Keywords:** cell differentiation, cell survival, dental enamel protein, enamel matrix proteins, gingiva, osteogenesis, stem cells

## Abstract

*Background and Objectives*: A derivative of the enamel matrix was used to speed up periodontal regeneration, including the formation of new cementum, alveolar bone, and periodontal ligament. In this study, human gingiva-derived stem cell–derived cell spheroids were used to assess the effects of an enamel matrix derivative on cell viability, osteogenic differentiation, and mineralization. *Materials and Methods*: Human gingiva-derived stem cells were used to create spheroids, which were then coupled with unloaded control groups and an enamel matrix derivative at a final concentration of 2.7, 27, 270, and 2700 μg/mL. The morphological examination of the created stem cell spheroids took place on days 1, 3, 5, and 7. The Live/Dead Kit assay was used to determine the qualitative viability of cells on days 3 and 7. Using the Cell Counting Kit-8, the quantitative vitality of the cell spheroids was assessed on days 1, 3, and 5. On days 7 and 14, alkaline phosphatase activity assays and Alizarin Red S staining were carried out to examine the osteogenic differentiation of the cell spheroids. RUNX2 and COL1A1 expression levels on days 7 and 14 were determined using real-time polymerase chain reaction. *Results:* The added enamel matrix derivative at the tested concentrations did not significantly alter the morphology of the applied stem cells’ well-formed spheroids on day 1. On days 3 and 7, the majority of the spheroids’ cells fluoresced green while they were being cultivated. Alkaline phosphatase activity data revealed a substantial rise in the 2700 μg/mL group on day 7 when compared to the unloaded control (*p* < 0.05). On days 7 and 14, calcium deposits were distinctly seen in each group. In the 27 and 2700 μg/mL groups, the treatment with the enamel matrix derivative resulted in noticeably higher values for the Alizarin Red S staining (*p* < 0.05). qPCR results showed that adding an enamel matrix derivative to the culture of the 27 μg/mL group raised the level of RUNX2 mRNA expression. *Conclusions:* These results lead us to the conclusion that a derivative of the enamel matrix may be used to promote osteogenic differentiation in stem cell spheroids.

## 1. Introduction

Amelogenin and ameloblastin make up most of the enamel matrix derivative made from porcine enamel matrix [1]. Under physiological conditions of a neutral pH and body temperature, the viscosity of the enamel matrix derivative diminishes, resulting in the precipitation of the enamel matrix derivative. At an acidic pH, a derivative of the enamel matrix can be dissolved in propylene glycol alginate [2]. Additionally, it should be noted that the enamel matrix derivative reportedly lingers for up to two weeks at the application site [3]. It has been demonstrated that a derivative of the enamel matrix is osteoinductive [4]. According to reports, using an enamel matrix derivative causes human bone cells to express more genes related to extracellular matrix production [5].

Periodontal tissues have been shown to respond to enamel matrix derivatives in a regenerative manner, and amelogenin or ameloblastin components help to partially recreate this response [6]. Increased blood vessel production following topical administration of an enamel matrix derivative to the soft tissues surrounding implants showed the substance might have a positive impact on wound healing [7]. Additionally, a product of the enamel matrix has been shown to promote endothelial cell migration and proliferation, which in turn increases angiogenesis [8,9]. It was shown that an enamel matrix derivative significantly enhanced the expression of interleukin-1 and the receptor activator of nuclear factor kappa-B ligand while significantly boosting the expression of prostaglandin E2 and osteoprotegerin [9].

These regeneration processes may be aided by stem cells [10]. Because of their capacity for self-renewal, propensity for multi-lineage differentiation, and immunomodulatory properties, mesenchymal stem cells are fascinating candidates for the cellular treatment of numerous organs [11]. Despite mesenchymal stem cells’ ability to function well, the therapeutic potential of these cells may be greatly hindered by the gradual loss of stem cell properties that happens with repeated passages [11]. Small adipose-derived stem cell spheroids that were cultivated in three dimensions without a scaffold persisted in in vitro and in vivo settings and encouraged bone healing [12]. As a stem cell–based biomaterial therapy, neurosphere-medium spheroids should offer excellent clinical potential for bone and tissue regenerative therapies [11]. 

To scale up and carry out medication cytotoxicity and efficacy testing as well as tissue engineering techniques, three-dimensional cellular aggregates must be created through controlled and reproducible processes [13]. The microenvironment of tissues and organs in nature can be mimicked by these aggregates [13]. Spheroids of bone marrow stromal cells and dental pulp stem cells, with good cell viability and average diameters of around 253 and 220 μm, respectively, were made using the equipment with the form that was thought to be most appropriate [13]. The primary objective of this study was to evaluate the effects of an enamel matrix derivative on the viability, osteogenic differentiation, and mineralization of human mesenchymal stem cell–derived cell spheroids. The secondary objectives were to determine the optimal concentration of the enamel matrix derivative for enhancing osteogenic differentiation and to investigate the underlying mechanisms by which the enamel matrix derivative influences the osteogenic differentiation.

## 2. Materials and Methods

### 2.1. Mesenchymal Stem Cells Obtained from Gingiva Used in the Design of the Current Investigation

**Figure 1:**
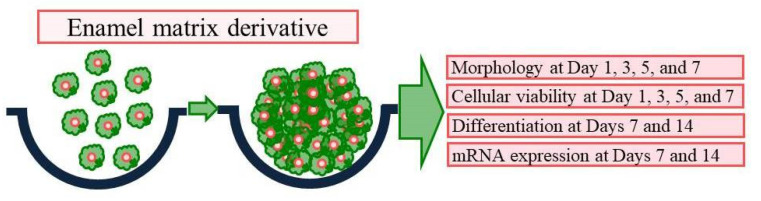
Study flow diagram for the overview.

An overview of the design of the current investigation is shown in Figure 1. The Institutional Review Board of Seoul St. Mary’s Hospital, College of Medicine, The Catholic University of Korea read the document and accepted the current study protocol (KC22SISE0184, Approval date: 5 April 2022 and KC22SISE0302, Approval date: 17 May 2022). The participant provided informed consent. The Declaration of Helsinki’s pertinent principles and rules were followed in all experiments. Every two to three days, the culture media was replaced. With 95% air and 5% CO_2_, the cells were cultivated in an incubator at 37 °C.

### 2.2. Manufacturing Stem Cell Spheroids

Following the previously described procedures, gingiva-derived mesenchymal stem cells (GMSCs) were obtained [14]. The tissues from the gingiva were de-epithelialized, diced, and enzyme-digested. The culture media was changed every two to three days, and the stem cells were put into a culture dish.

Stem cells were seeded at a density of 1 × 10^6^ cells/well on silicon elastomer-based concave microwells (StemFIT 3D; MicroFIT, Seongnam-si, Republic of Korea) and grown in an osteogenic medium. A commercially available enamel matrix derivative (Emdogain^®^, Straumann, Basel, Switzerland) was diluted with a final concentration of 2.7, 27, 270, and 2700 μg/mL. Every one to two days, a new medium was used in place of the old one. On days 1, 3, 5, and 7, morphological analysis was performed using an inverted microscope (CKX41SF, Olympus Corporation, Tokyo, Japan).

### 2.3. Assessing the Quality of Cell Vitality

Spheroids of stem cells were grown in osteogenic media with -MEM as the primary ingredient (Gibco, Grand Island, NY, USA). For the qualitative assessments of cellular viability on days 3 and 7, a commercially available two-color assay based on plasma membrane integrity and esterase activity (Live/Dead Kit assay, Molecular Probes, Eugene, OR, USA) was utilized [15]. Next, 2 µL of 50 mM calcein acetoxymethyl ester and 4 µL of 2 mM ethidium homodimer-1 were applied to the spheroids. The spheroids were incubated for 60 min at room temperature before being rinsed with growth medium. Then, using a fluorescent microscope (Axiovert 200; Zeiss, Göttingen, Germany), the stem cell spheroids were examined. 

### 2.4. Quantitative Analysis of Cell Vitality

On days 1, 3, 5, and 7, a quantitative cellular viability test was carried out utilizing an assay kit based on water-soluble tetrazolium salt (Cell Counting Kit-8, Dojindo, Tokyo, Japan) [16]. The cultures were supplemented with water-soluble tetrazolium salt, and the spheroids were incubated for 1 h at 37 °C. The assay, which measures the capacity of mitochondrial dehydrogenases to oxidize substances, was used to identify viable cells. Tetrazolium-8 was converted into a formazan product that is water soluble. A microplate reader was used to measure the spectrophotometric absorbance (BioTek, Winooski, VT, USA).

### 2.5. Alkaline Phosphatase Activity Levels and Calcium Deposition

On days 7 and 14, osteogenic differentiation was evaluated using an anthraquinone dye assay to measure calcium deposits and the degree of alkaline phosphatase activity [17]. On days 7 and 14, cell spheroids cultivated on culture plates with an osteogenic medium were obtained. Alkaline phosphatase activity was measured using a commercial kit (K412-500, BioVision, Inc., Milpitas, CA, USA). After combining the cell lysates with an assay solution (K412-500; BioVision, Inc.) and incubating it at 4 °C for 30 min, the absorbance at 405 nm was measured [16].

### 2.6. Real-Time Quantitative Polymerase Chain Reaction for Total RNA Extraction and Measurement of RUNX2 and COL1A1 mRNA (qPCR)

Following the manufacturer’s instructions, total RNA extraction was carried out using a commercial kit (Thermo Fisher Scientific, Inc., Waltham, MA, USA) [18]. Agilent Technologies’ RNA 6000 Nano Chip kit was used to assess RNA quality, and a spectrophotometer was used to assess RNA amount by measuring the ratio of absorbance at 260 nm and 280 nm (ND-2000, Thermo Fisher Scientific, Inc.). Reverse transcriptase was utilized with RNA as a template for reverse transcription (SuperScript II; Invitrogen, Carlsbad, CA, USA).

On day 14, qPCR identified mRNA expression. To create the sense and antisense PCR primers, we used GenBank. The information about the primers used in this study is displayed in Table 1. Utilizing the housekeeping gene β-actin, normalization was carried out. Following the instructions provided by the manufacturer, real-time PCR was carried out on the PCR System (StepOnePlusTM; Applied Biosystems) using the SYBR Green PCR Kit (Applied Biosystems, Waltham, MA, USA) [19,20].

### 2.7. Statistical Evaluation

Each value is displayed as the mean minus the standard deviation. Testing for normality and variance equality was performed. The one-way analysis of variance with Tukey’s post hoc test was used to compare the groups (SPSS 12 for Windows, SPSS Inc., Chicago, IL, USA). For each analysis, three experimental replicates were assessed.

## 3. Results

### 3.1. Spheroids of Mesenchymal Stem Cells from Human Gingiva

**Figure 2:**
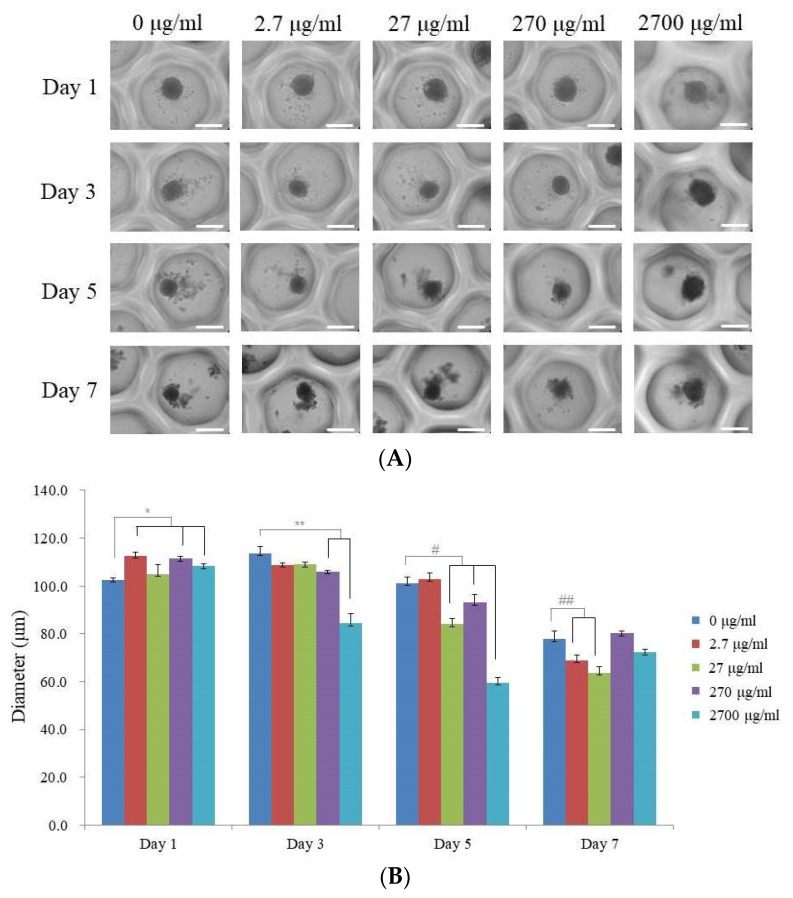
Analysis of the morphology. (**A**) On days 1, 3, 5, and 7, the morphologies of the stem cell spheroids exposed to various enamel matrix derivative concentrations were examined. The scale bar represents 200 μm (original magnification: 200). (**B**) The stem cell spheroids’ sizes on days 1, 3, 5, and 7. * *p* < 0.05 on day 1 compared to the time-matched unloaded control group. ** *p* < 0.05 on day 3 compared to the time-matched unloaded control group. # *p* < 0.05 on day 7 compared to the time-matched unloaded control group. ## *p* < 0.05 on day 14, compared to the time-matched, unloaded control group.

Figure 2A depicts on days 1, 3, 5, and 7 the morphology of the spheroids treated with enamel matrix derivative, those not treated, and the groups with the final dilution of 0.001, 0.01, 0.1, and 1. Over the course of seven days, the stem cell spheroids exhibited no morphological alterations. While the size of each stem cell spheroid changed from day 1 to day 7, they all retained their round shape. Figure 2B displays the spheroids’ diameters on days 1, 3, 5, and 7. For the groups with 0, 2.7, 27, 270, and 2700 μg/mL on day 1, the diameters were 102.5 ± 0.8, 112.6 ± 1.4, 105.0 ± 4.0, 111.4 ± 1.0, and 108.3 ± 0.9 µm, respectively (*p* < 0.05). On day 3, the diameters of the 0, 2.7, 27, 270, and 2700 μg/mL groups were 113.7 ± 2.7, 108.9 ± 0.6, 109.0 ± 0.8, 106.1 ± 0.3, and 84.3 ± 4.2 µm, respectively (*p* < 0.05). On day 5, the diameters of the 0, 2.7, 27, 270, and 2700 μg/mL groups were 101.2 ± 2.4, 102.9 ± 2.5, 84.0 ± 2.3, 93.0 ± 3.3, and 59.6 ± 2.0 µm, respectively (*p* < 0.05). On day 7, the diameters of the 0, 2.7, 27, 270, and 2700 μg/mL groups were 77.8 ± 3.5, 69.0 ± 2.1, 63.7 ± 2.4, 80.2 ± 0.8, and 72.2 ± 1.2 µm, respectively (*p* < 0.05).

### 3.2. Determining the Vitality of Cells Qualitatively and Values in Numbers for Cellular Viability

**Figure 3:**
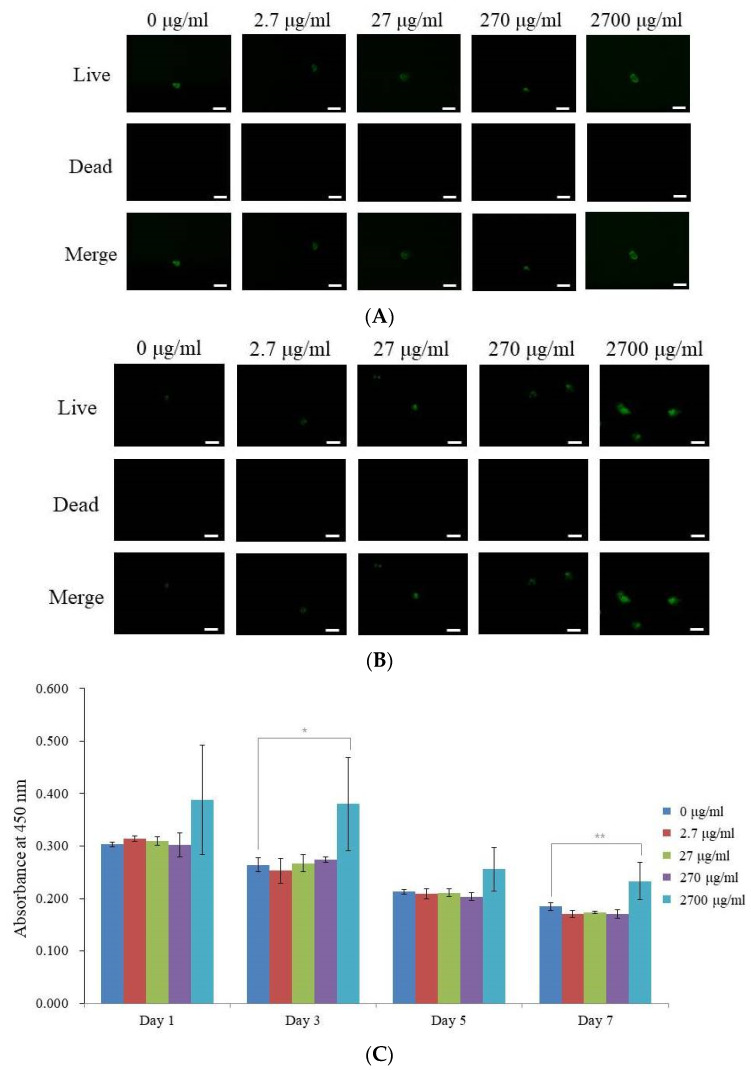
Cellular viability testing. The information about the primers used in this study is displayed in Table 1. (**A**) On day 3, the stem cell spheroids’ live, dead, and combined cell pictures were examined. (**B**) On day 7, the stem cell spheroids were imaged as live, dead, and merged cells. The scale bar represents 200 μm (original magnification: 100). (**C**) On days 1, 3, 5, and 7, we determined the cell viability with the Cell Counting Kit-8. * On day 3, *p* < 0.05 compared to the time-matched unloaded control group. ** On day 7, *p* < 0.05 compared to the time-matched unloaded control group.

On days 3 and 7, the qualitative vitality of the stem cells was examined using a Live/Dead Kit assay (Figure 3A,B). On day 3, we could see that the majority of the stem cells had a rounded form and bright green fluorescence, indicating that they were alive (Figure 3A). The cells on day 7 had incubated for longer but the green fluorescence did not alter noticeably (Figure 3B).

In Figure 3C, the quantitative cellular viability for days 1, 3, 5, and 7 is depicted. On day 1, the absorbance values at 450 nm for the enamel matrix derivative in the 0, 2.7, 27, 270, and 2700 μg/mL groups were 0.303 ± 0.005, 0.314 ± 0.005, 0.309 ± 0.008, 0.302 ± 0.023, and 0.388 ± 0.104, respectively (*p* > 0.05). On day 3, the absorbance values at 450 nm for the enamel matrix derivative in the 0, 2.7, 27, 270, and 2700 μg/mL groups were 0.264 ± 0.013, 0.253 ± 0.024, 0.267 ± 0.017, 0.274 ± 0.006, and 0.380 ± 0.089, respectively (*p* < 0.05). On day 5, the absorbance values at 450 nm for the enamel matrix derivative in the 0, 2.7, 27, 270, and 2700 μg/mL groups were 0.213 ± 0.005, 0.209 ± 0.009, 0.211 ± 0.007, 0.204 ± 0.007, and 0.256 ± 0.042, respectively (*p* > 0.05). On day 7, the absorbance values at 450 nm for the enamel matrix derivative in the 0, 2.7, 27, 270, and 2700 μg/mL groups were 0.185 ± 0.008, 0.171 ± 0.007, 0.174 ± 0.003, 0.171 ± 0.009, and 0.233 ± 0.036 (*p* < 0.05), respectively.

### 3.3. Analyzing the Activity of Alkaline Phosphatase with Alizarin Red S Staining

**Figure 4:**
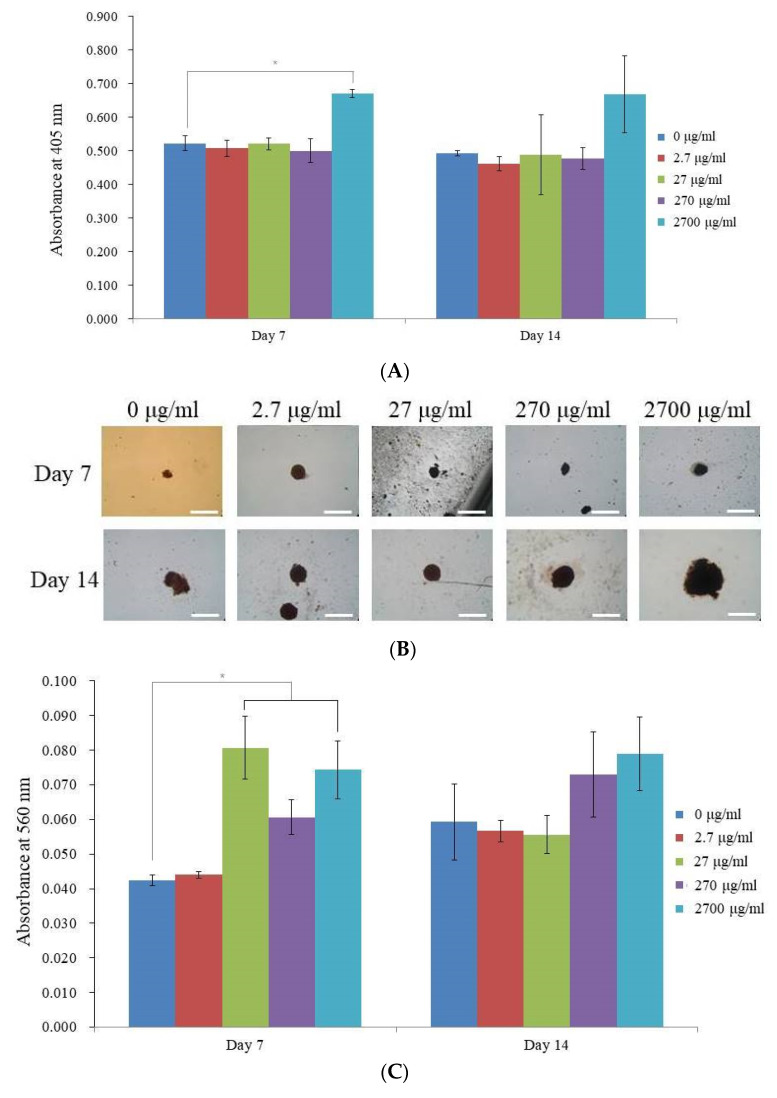
Osteogenic differentiation. (**A**) Results of the alkaline phosphatase activity tests on days 7 and 14 are shown graphically. * *p* < 0.05 in comparison to the time-matched, unloaded control group. (**B**) Alizarin Red S staining on days 7 and 14. (**C**) Analysis of Alizarin Red S staining in numbers on days 7 and 14. * *p* < 0.05 compared to the time-matched 0 group.

The absorbance values for the enamel matrix derivative for the 0, 2.7, 27, 270, and 2700 μg/mL groups on day 7 were 0.523 ± 0.022, 0.508 ± 0.025, 0.521 ± 0.019, 0.501 ± 0.035, and 0.671 ± 0.012, respectively (*p* < 0.05). When compared to the unloaded control group on day 7, the alkaline phosphatase activity results for the 2700 μg/mL group indicated a significant increase (*p* < 0.05) (Figure 4A). The absorbance values for the enamel matrix derivative in the 0, 2.7, 27, 270, and 2700 μg/mL groups on day 14 were 0.492 ± 0.008, 0.462 ± 0.021, 0.489 ± 0.120, 0.477 ± 0.032, and 0.669 ± 0.115, respectively (*p* > 0.05).

On days 7 and 14, calcium deposits were plainly visible in each group (Figure 4B). For the 0, 2.7, 27, 270, and 2700 μg/mL groups, the relative values for Alizarin Red S staining on day 7 were 100.0 ± 3.6, 103.9 ± 2.4, 190.6 ± 21.4, 143.3 ± 11.7, and 175.6 ± 19.8%, respectively (*p* < 0.05) (Figure 4C). For the 27, 270, and 2700 groups, the treatment with the enamel matrix derivative resulted in noticeably higher values for the Alizarin Red S staining (*p* < 0.05). For the 0, 2.7, 27, 270, and 2700 μg/mL groups, the relative values for Alizarin Red S staining on day 14 were 140.2 ± 25.9, 133.9 ± 7.2, 131.5 ± 13.0, 172.4 ± 29.0, and 186.6 ± 25.0%, respectively (*p* > 0.05). In the 270 and 2700 μg/mL groups, relative higher values were seen, but this did not reach statistical significance (*p* > 0.05).

### 3.4. qPCR Analysis of RUNX2 and COL1A1

**Figure 5:**
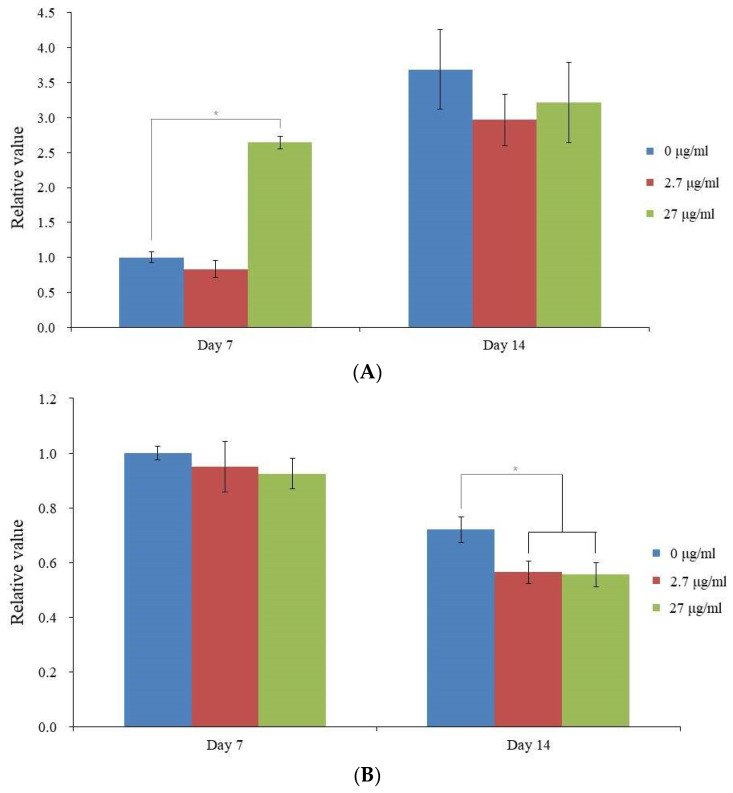
Expression of mRNA in quantitative form. (**A**) Real-time polymerase chain reaction measurement of RUNX2 mRNA expression on days 7 and 14. On day 7, * *p* < 0.05 in comparison to the unloaded control group. (**B**) Real-time polymerase chain reaction measurement of COL1A1 mRNA expression on days 7 and 14. Day 14: * *p* < 0.05 in comparison to the unloaded control group.

According to qPCR, the mRNA levels of RUNX2 for the 0, 2.7, and 27 μg/mL groups were 1.000 ± 0.081, 0.834 ± 0.128, and 2.643 ± 0.085, respectively, on day 7 (Figure 5A). Vitamin E supplementation significantly increased RUNX2 expression at 0.01 (*p* < 0.05). According to qPCR, the mRNA levels of COL1A1 for the 0, 2.7, and 27 μg/mL groups were 1.000 ± 0.024, 0.950 ± 0.093, and 0.924 ± 0.055, respectively (Figure 5B).

## 4. Discussion

This study used spheroid culture to examine how an enamel matrix derivative affected cell survival, osteogenic differentiation, and mineralization. Alkaline phosphatase activity, mineralization tests, and RUNX2 mRNA levels all indicated differentiation into an osteogenic lineage.

Utilizing a variety of materials, successful tissue regeneration was accomplished [15,21]. The use and indications for enamel matrix derivatives are constantly expanding [22]. An enamel matrix derivative was employed to hasten periodontal regeneration, including the growth of new cementum, alveolar bone, and periodontal ligament [23]. An earlier study discovered that enamel matrix derivative was superior to open flap debridement for the treatment of intrabony dental lesions [24]. Enamel matrix derivative is used with bone graft material to help heal severe intrabony defects by repairing wounded tissue [25]. In a previous study, bone graft material was combined with an enamel matrix, which was said to have effects similar to those of recombinant human platelet-derived growth factor-BB when used to successfully heal intrabony defects [26]. A previous study indicated that the adjunct combination of the two improved the clinical and radiographic outcomes in intrabony defects when one- to two-wall periodontal defects were treated with either a bone substitute or an enamel matrix derivative alone [27]. A bone substitute and enamel matrix derivative were used to repair the buccal plate extraction socket [28]. Additionally, it was hypothesized that using an enamel matrix derivative could help patients whose inflammatory processes were fast progressing and causing the degeneration of periodontal tissue [29]. Additionally, severe intrabony flaws linked with the palatal radicular groove were successfully treated without endodontic therapy or retreatment by applying an enamel matrix derivative [30].

Dental implants are just one application for enamel matrix derivatives. Increased stem cell proliferation and osteogenic differentiation were brought about by the application of an enamel matrix derivative to titanium surfaces, and endothelial cell expression of angiogenic genes was also improved [31,32]. More soft tissue showed faster healing after the titanium implant surface was covered with an enamel matrix derivative [33]. The addition of an enamel matrix derivative to the mechanical debridement of peri-implant mucositis was proposed [34]. Additionally, peri-implantitis was successfully treated using hydroxyapatite produced from cows and an enamel matrix derivative [35]. Soft tissue surgery has also used enamel matrix derivatives [36,37]. In more recent years, a coronally advanced flap was used to recover the gingival recessions in combination with an enamel matrix derivative [36]. A recent study found that using the modified tunnel approach on numerous recession flaws produced clinically and aesthetically excellent results with an enamel matrix derivative [37].

Spheroid cultures were used in this investigation. Spheroids and newly extracted primary tissues have much lower quantities of glycolysis intermediates, amino acids, and lipids than traditionally cultivated cells, which express higher levels of these molecules [38]. Spheroids also start to specialize and produce metabolites that are specific to the tissue from which they originated. It is advised that 3D culture techniques be used instead of animal testing because three-dimensionally produced cells exhibit significant metabolic similarities to the original tissue [38]. Due to their geometric nature, spheroids are known to have serious flaws [39]. Diffusion is the only method used by cells to move nutrients or get rid of waste [39]. Spheroids’ inner contents thus lack nutrition and oxygen and retain CO_2_, which leads to necrosis, whereas the outer layer’s cells efficiently transmit nutrients and eliminate waste [39]. Slowing inner cell growth and a rise in the cell death rate are drawbacks of 3D cultivation techniques for mesenchymal stem cells [40]. To combat this, the multilineage differentiation potential and stemness qualities of the 3D spheroids based on a self-feeder layer have been demonstrated [41]. Spheroids created with higher cell densities were larger than those formed with lower cell densities, and they also had a larger necrotic core surrounded by more quiescent cells and were resistant to pharmacological therapy [42].

In this investigation, gingival stem cells were employed. In a recent study, it was shown that the right kind of stem cells is essential for neuromuscular regeneration and that neural crest stem cells have a specific advantage and an ability to treat peripheral nerve injuries by promoting re-innervation [10]. Due to their capacity for regeneration, adipose-derived stem cells are helpful in treating bone and cartilage abnormalities [12]. The fundamental mechanism was examined previously. Mitogen-activated protein kinase (MAPK) and Wnt/beta-catenin signaling pathways have been shown to interact [43]. The canonical Wnt/beta-catenin pathway is essential for healthy embryonic development; mutations and abnormal expressions of certain of its constituent parts can cause cancer. The Wnt/beta-catenin signaling has been found to be impacted by mitogen-activated protein kinase pathways, which are prominent in intracellular signaling [43]. Through activating the Wnt/beta-catenin pathway, an enamel matrix derivative improved BMSCs’ osteogenic differentiation in a prior work [44]. Dental pulp stem cells were encouraged to differentiate into odontoblasts by an enamel matrix derivative via activating MAPK signaling pathways [45].

Given the constraints, the impacts of the enamel matrix derivative can be improved. Prior to now, it was proposed that the enamel matrix derivative, despite its limitations as a result of its gel-like consistency, can be utilized alone in periodontal regeneration, particularly in non-self-supporting defects [46]. According to a recent publication, treatment of partially contained defects with an enamel matrix derivative alone or in combination with deproteinized bovine bone mineral resulted in equivalent clinical and radiographic outcomes after 12 months [47]. All the defects displayed satisfactory clinical and radiographic outcomes, according to the two-year follow-up study comparing the effects of the enamel matrix derivative with particulate autogenous bone in the treatment of non-contained intrabony defects [48]. After at least five years of monitoring, the intrabony defects repaired with enamel matrix derivative’s reentry measures revealed stable outcomes in five out of seven patients [49]. Similar to this, a recent publication showed that a tooth affected at the apex could be successfully restored using enamel matrix derivative [50].

There may be some limitations to this study. The study used cell cultures, which might not have accurately reflected how cells behave in animals or humans [51]. Secondly, the study examined only a small subset of enamel matrix derivative concentrations, which may not accurately reflect the full range of effective amounts [52]. The study tracked cell behavior for up to 14 days only, which may not accurately represent how the enamel matrix derivative will affect the cells over the long run [53]. It would also be helpful to investigate the effectiveness of enamel matrix derivative at various concentrations and exposure times. Further studies should include in vivo investigations to evaluate the outcomes of the in vitro tests and to ascertain the clinical relevance and potential of enamel matrix derivative in enhancing tissue regeneration. By conducting long-term research to evaluate the stability of the regenerated tissues, it may be possible to better comprehend the potential applications of this approach.

## 5. Conclusions

Alkaline phosphatase activity and mRNA expression showed that the application of the enamel matrix derivative promoted differentiation while preserving cell viability. These results lead us to the conclusion that a derivative of the enamel matrix may be used to promote osteogenic differentiation in stem cell spheroids.

## Figures and Tables

**Table 1:** Information on primers used in this study.

Primer	Accession No.	Sequence
RUNX2	NM_001015051.3	forward: 5′-CAGTTCCCAAGCATTTCATCC-3′ reverse: 5′-AGGTGGCTGGATAGTGCATT-3′
COL1A1	NM_000088.4	forward: 5′-TACCCCACTCAGCCCAGTGT-3′ reverse: 5′-CCGAACCAGACATGCCTCTT-3′
β-actin	NM 001101	forward: 5′-AATGCTTCTAGGCGGACTATGA-3′ reverse: 5′-TTTCTGCGCAAGTTAGGTTTT-3′

## Data Availability

This publication contains all of the data that were created or examined during this study.
